# Atomic Force Microscopy Study of Protein–Protein Interactions in the Cytochrome CYP11A1 (P450scc)-Containing Steroid Hydroxylase System

**DOI:** 10.1007/s11671-010-9809-5

**Published:** 2010-09-30

**Authors:** YD Ivanov, PA Frantsuzov, A Zöllner, NV Medvedeva, AI Archakov, W Reinle, R Bernhardt

**Affiliations:** 1 Institute of Biomedical Chemistry RAMS, Pogodinskaya st. 10, 119121, Moscow, Russia; 2 Saarland University, Saarbrücken, Germany

**Keywords:** Atomic force microscopy, Photon correlation spectroscopy, Cytochrome CYP11A1, Monomerization, Complex formation

## Abstract

Atomic force microscopy (AFM) and photon correlation spectroscopy (PCS) were used for monitoring of the procedure for cytochrome CYP11A1 monomerization in solution without phospholipids. It was shown that the incubation of 100 μM CYP11A1 with 12% Emulgen 913 in 50 mM KP, pH 7.4, for 10 min at *T* = 22°C leads to dissociation of hemoprotein aggregates to monomers with the monomerization degree of (82 ± 4)%. Following the monomerization procedure, CYP11A1 remained functionally active. AFM was employed to detect and visualize the isolated proteins as well as complexes formed between the components of the cytochrome CYP11A1-dependent steroid hydroxylase system. Both Ad and AdR were present in solution as monomers. The typical heights of the monomeric AdR, Ad and CYP11A1 images were measured by AFM and were found to correspond to the sizes 1.6 ± 0.2 nm, 1.0 ± 0.2 nm and 1.8 ± 0.2 nm, respectively. The binary Ad/AdR and AdR/CYP11A1_mon_ complexes with the heights 2.2 ± 0.2 nm and 2.8 ± 0.2 nm, respectively, were registered by use of AFM. The Ad/CYP11A1_mon_ complex formation reaction was kinetically characterized based on optical biosensor data. In addition, the ternary AdR/Ad/CYP11A1 complexes with a typical height of 4 ± 1 nm were AFM registered.

## Introduction

Hemeproteins belonging to cytochrome P450 superfamily play an important role in metabolism of a broad spectrum of endogenous and exogenous chemicals [1]. CYP11A1-dependent monooxygenase system is responsible for cholesterol conversion to pregnenolone [2,3]. The electron transfer chain of this system includes adrenodoxin reductase (AdR), adrenodoxin (Ad) and CYP11A1. AdR transfers electrons from NADPH to CYP11A1 via Ad [4]. CYP11A1-dependent monooxygenase system is unique in its organization. This is a mixed-type system since electron transfer components Ad and AdR are water-soluble proteins, while CYP11A1 is a membrane-bound hemeprotein [5]. To gain a better insight into the intrinsic mechanism of electron transfer in this system, it is necessary to have information on the structure and properties of individual proteins and their complexes. At present, the crystal structure of Ad is already solved [6], and the size of the ferredoxin molecule is determined (3.8 × 3.4 × 4.4 nm). The crystal structure of AdR has also been solved, its size being equal to 5.8 × 5.4 × 4.0 nm [7]. As is known, the isolated membrane cytochrome CYP11A1 is able to form oligomers in solution [8]. Therefore, the structure of CYP11A1 still remains to be clarified. No NMR or X-ray data for this protein have as yet been obtained.

Only the data on the structure of the cross-linked AdR/Ad complex has so far been reported [9]. The structure of complexes that are formed within CYP11A1 system in native conditions is yet to be clarified. The size of this complex equals 7.4 × 7.0 × 13.3 nm. It is known that the components of CYP11A1-dependent monooxygenase system can form binary complexes, as has been shown using different approaches: NMR [9], spectroscopy [10], optical biosensor [10-12], chemical cross-linking [13,14] and isothermic calorimetry [15]. Moreover, the formation of ternary complexes between Ad, AdR and CYP11A1 has been registered in gel-filtration [16] and optico-biosensoric studies [17].

Atomic force microscopy (AFM) method is finding increasing application in structural characterization of proteins in native conditions. This method was successfully used to study the water-soluble cytochrome P450cam system [18]. To simplify the modeling of the electron transfer chain of the cytochrome P450cam system, it was reconstituted in solution as was reported in [18]. The AFM investigation of membrane-bound cytochrome P450 systems is complicated by the presence of phospholipid membrane in their constituent proteins. It is known that membrane proteins are able to form aggregates upon solubilization. This hampers the analysis of their complexes. The most convenient approach to overcome this difficulty is based on the modeling of membrane-bound P450 system in solution containing a detergent (instead of phospholipid membranes), as was proposed for the cytochrome P4502B4 system [19]. This approach was successfully applied to AFM visualization of binary and ternary complexes of proteins involved in electron transfer chain within the membrane P450 2B4 system [20,21].

In this paper, a similar approach was developed for AFM visualization of proteins and their complexes within the mixed-type CYP11A1 system. For this purpose, CYP11A1 monomerization was carried out in the presence of Emulgen 913. It was shown that CYP11A1 is predominantly present in a monomeric form after monomerization procedure. The protein's monomerization degree was controlled via AFM and PCS. The functional activity of the monomerized CYP11A1 thus obtained was demonstrated. Furthermore, it was shown that solubilized Ad and AdR are predominantly present in their monomeric forms as well. The AFM application allowed to visualize and measure the heights of the individual proteins AdR, Ad, CYP11A1 as well as binary AdR/Ad and AdR/CYP11A1 complexes. Moreover, the formation of ternary AdR/Ad/CYP11A1 complexes was registered in CYP11A1 system.

## Materials and Methods

### Chemicals

Emulgen 913 was purchased from Kao Atlas (Osaka, Japan); all other chemicals were from Reakhim (Moscow, Russia). Ultrapure water was obtained using the Milli-Q system (Millipore, Bedford, USA).

### Protein Expression and Purification

Bacteria were grown as previously reported [22] with slight modifications. Briefly, we used freshly transformed E. coli BL21DE3 to inoculate a preculture. The bacteria were allowed to grow in ampicillin-containing nutrient broth medium at 37°C overnight. These cultures were used to inoculate 4 l of a main culture containing ampicillin. Isopropyl-1-thio-D-galactopyranoside was added to induce heterologous protein production, and afterward cultures were grown at 37°C for 16 h. Recombinant Ad was purified after sonification as described, and the final concentration of Ad was determined using ε 414 = 9.8 mM^-1^ cm^-1 ^[23]. The purity of the Ad preparation was estimated by determining the relative absorbance of the protein at 414 and 273 nm, i.e. its Q value (A414/A273). AdR was heterologously expressed and purified as described elsewhere [24]. The molar extinction coefficient used for estimation of AdR concentration was ε 450 = 10.9 mM^-1^ cm^-1 ^[25]. Isolation of CYP11A1 from bovine adrenal glands was performed as previously described [26]. CYP11A1 concentration was estimated by carbon monoxide difference spectra using ε (450–490) = 91 (mM cm)^-1^.

### Procedure for CYP11A1 Monomerization

For monomerization of cytochrome CYP11A1, the detergent Emulgen 913 in the concentration range 4–12% was chosen. The monomerization scheme was as follows: to 2 μl of stock solution of CYP11A1 (100 μM) in 50 mM KP, pH 7.4, were added 1.3 μl of Emulgen 913 at three various concentrations (10%, or 20%, or 30% solution) at *T* = 22°C. The final concentrations of Emulgen 913 in the three incubation solutions were 4, 8 and 12%, respectively. The mixture obtained was incubated at room temperature (22°C) for 10 min.

### AFM Experiments and Samples' Preparation

AFM experiments were carried out using the direct surface adsorption method [27]. As support, the mica was used.

For visualization of individual non-monomerized and monomerized CYP11A1 protein molecules, the appropriate protein solution was diluted in 50 mM K-phosphate buffer, pH 7.4 (50 KP) to obtain 1 μM protein concentration; 5 μl of obtained solution were immediately deposited onto the freshly cleaved mica surface and left for 3 min. For visualization of the individual Ad and AdR protein molecules, 5 μl of 1.0 μM solution of an appropriate protein in 50 mM K-phosphate buffer, pH 7.4, were deposited onto the freshly cleaved mica surface and left for 3 min. After that, each sample was first rinsed with the same buffer, then with ultrapure distilled water and dried in airflow. The binary complexes were obtained by mixing 10 μl of 5 μM solutions of appropriate individual proteins in 50 KP, pH 7.4. Then, the mixture was incubated for 10 min, diluted 2.5 times in the same buffer, and a 5-μl portion of the mixture was immediately placed onto mica. The ternary complexes were obtained by mixing 10 μl of 7.5 μM solutions of appropriate individual proteins in 50 KP, pH 7.4. Then the mixture was incubated for 10 min, diluted 2.5 times in the same buffer, and a 5-μl portion of the mixture was immediately placed onto mica. As was shown in an earlier research [28], with relative humidity exceeding 45%, the mica surface is covered with a water layer. Therefore, in the present study all the measurements were carried out at room temperature and at 60–70% air humidity, the protein molecules under study remained hydrated throughout. The choice of protein concentration was dictated by inherent limitations of the AFM technique: at higher concentrations, the molecules under observation formed layers on the mica support, which excluded the identification of individual objects.

All AFM experiments were carried out in a tapping mode on a multimode "NTEGRA" atomic force microscope (NT-MDT, Moscow, Russia) in air. Cantilevers NSG 10 produced by "NT-MDT" (Russia) were used. The resonant frequency of the cantilevers was 190–325 kHz, and the force constant was about 5.5–22.5 N/m. The calibration of the microscope by height was carried out on a *TGZ1* calibration grating (NT-MDT, Moscow, Russia) with the step height 22 ± 0.5 nm. The supersharp probes with the radius of curvature of about 1–3 nm were used for measuring of CYP11A1 monomers' volumes. As supersharp probes, NSG01_DLC microprobes (NT-MDT, Russia) with a typical resonant frequency of 115–190 kHz were used.

The total number of measured particles in each sample was not less than 600, and the number of measurements for each sample was no less than 16, i.e. there were 4 measurements in each of the four series.

### Analysis of AFM Images

The density of protein distribution with height, ρ(*h*), was calculated as ρ(*h*) = (*N*_*h*_*/N*) × 100%, where *N*_*h*_ is the number of imaged proteins with height *h*, and *N* is the total number of imaged proteins. The calculation was carried out using a step of 0.2 nm.

To calculate the deaggregation degree, the dependence of distribution density ρ(*h*) of CYP11A1 images with height (*h*) was constructed:

(1) $$\rho \left(h\right)=\left(N_{h}/N\right)\times 100\%.$$

The dependence of this distribution was approximated using root-mean square method by the sum of two curves:

(2) ρ(h)=ρ1(h)+ρ2(h)=∑i=12Ki×(h−mi)2bi2×EXP[−(h−mi)22bi2]

where *K*_*i*_, *m*_*i*_, *b*_*i*_ are the parameters of ρ(*h*_*i*_) distribution. The maximum of ρ_*i*_(*h*) was calculated from Eq. (2).

For the analysis of distribution with heights and volumes (ρ(*h*,*V*)) of imaged CYP11A1, (ρ(*h*,*V*)) was calculated as

(3) $$\rho \left(h,V\right)=\left(N_{h,V}/N\right)\times 100\%,$$

where *N*_*h,V*_ is the number of imaged proteins with the height *h*, and the volume *V*.

Values of height maximums and distributions widths, represented in text, were calculated from Eq. 2.

### PCS Measurements

Photon correlation spectroscopy (PCS) measurements were carried out by use of N5 Submicron Particle Size Analyzer (Beckman Coulter, Inc). The principle of registration is based on measuring the interference pattern of light scattered on particles in solution by use of photon correlation spectroscopy (PCS). Measurements were made at the light-scattering angle of 90°. Protein solution (the stock one or the one subjected to monomerization procedure) was diluted in 50 mM KP, pH = 7.4, and placed into the measuring cuvette of Analyzer. Protein concentration was so selected as to make the intensity of dissipated light at 90° not lower than the sensitivity threshold corresponding to 5 × 10^4^ counts. CYP11A1 and AdR concentrations were 5 μM for each protein. For Ad, the concentration was 0.2 mM. The measurements were made up to the accumulation of the signal during 200 s.

The calibration of the correlometer was performed using the set of latexes with the diameters 40, 50, 150 and 500 nm and the cytochrome C (2.9 × 5.5 × 2.3 nm) with the known X-ray structure from PDB [29]. In this size range, the measured sizes of latex corresponded to nominal with a root-mean square deviation of 10%.

### Optical Biosensor Measurements

Formation of the complex between monomeric CYP11A1 and Ad was additionally assayed on a Biacore 3000 system, using the optical biosensor method as described before with slight modifications [30,31].

Briefly, after activation of the CM5 chip with *N*-ethyl-*N'*-dimethylaminopropyl-carbodiimide (EDC) and *N*-hydroxysuccinimide (NHS), 75 μL of a 200 μM Ad solution was injected with a flow of 5 μl min^-1^ at 20°C. The immobilization procedure was completed by injecting 1 M ethanolamine hydrochloride in order to block the remaining ester groups. Approximately 400 RU (response units) Ad was immobilized on the dextran matrix. In order to match the experimental conditions employed for the AFM measurements, we used a 50 mM potassium phosphate buffer (pH 7.4) containing 1% Emulgen 913. Binding of monomeric or oligomeric CYP11A1 to immobilized Ad was analyzed by injecting CYP11A1 solutions with concentrations varying between 1 and 100 nM. Each concentration was injected at least three times. To visualize unspecific background interactions between the dextran matrix and CYP11A1, a reference cell (i.e. the cell without Ad) was created. Ten microliters of 1 mM NaOH was used as regeneration solution. *K*_*D*_ values were determined using the software Biaeval 4.1. Averaged binding curves for the interaction between Ad and varying CYP11A1 concentrations were fitted simultaneously using the 1:1 Langmuir-binding model. *K*_*D*_ values were determined from the fit with the lowest standard deviation.

### Control of Functionality of Monomeric CYP11A1

These assays were aimed toward demonstrating the functionality of monomeric CYP11A1. For this purpose, we investigated the conversion of 7-dehydrocholesterol to 7-dehydropregnenolone cortisol [32] using monomeric CYP11A1. In vitro reconstitution assays were performed as described before [33] with slight modifications. Briefly, the reaction mixture (0.5 ml) consisted of either CYP11A1 (0.4 μM) that has been monomerized using Emulgen 913 as described earlier or oligomeric CYP11A1 (0.4 μM), AdR (0.5 μM), Ad (0 to 4 μM), 7-dehydrocholesterol (400 μM) and MgCl_2_ (1 mM) in 50 mM potassium phosphate buffer (pH 7.4) containing 0.05% (v/v) Tween 20.

Substrate conversion was started by the addition *of NADPH* up to the final concentration of 100 μM. In addition to this, glucose-6-phosphate (5 μM) and glucose-6-phosphate dehydrogenase (1 U) were added to the reaction mixture. After the reaction was completed, steroids were extracted with chloroform and then separated on a Jasco reversed-phase HPLC system of the LC900 series using a 3.9 × 150 mm Waters Nova-Pak C18 column at 40°C. The mobile phase used for the separation was a mixture of acetonitrile/2-propanol (30:1). Product quantification was performed by correlating the product peak integrals with the peak area of a known internal standard (5 nmol cortisol) that was added prior to the chloroform extraction. *K*_*m*_ and *V*_max_ values were determined by plotting the substrate conversion velocity versus Ad concentration and applying the Michaelis–Menten kinetics (hyperbolic fit) using the program SigmaPlot 2001. Each experiment was performed four times. The velocity of the Ad-dependent product formation was expressed in nmol product × min^-1^ × nmol CYP11A1^-1^.

### Analytical Methods

Proteins were analyzed via SDS gel electrophoresis in order to detect major impurities in protein preparations. The results obtained from these measurements revealed no impurities in the purified protein samples of all three components of the CYP11A1 electron transfer chain (data not shown).

In order to check possible structural changes in the protein conformations of the monomerized and oligomeric proteins, UV/VIS and CD spectroscopy have been performed.

Absorption spectra in the UV/VIS region (250–700 nm) were recorded at room temperature on a double-beam spectrophotometer UV2101PC (Shimadzu; Kyoto, Japan). UV/VIS spectra of monomeric or oligomeric proteins revealed no significant changes (data not shown). UV/VIS spectra of CYP11A1 displayed a pronounced peak at 392 nm, indicating that the protein is in its high spin conformation. Carbon monoxide difference spectroscopy performed for CYP11A1 displayed a pronounced peak at 450 nm, whereas the peak at 420 nm (non-functional protein) was not observable.

CD spectra of oxidized monomeric and oligomeric CYP 11A1 were recorded on a Jasco 715 spectropolarimeter as described before [34]. All protein samples were diluted in 10 mM KP (pH 7.4). Possible changes in the secondary structures of the proteins were investigated by recording CD spectra in the range of 195–260 nm. CD measurements in the 250–650 nm range were performed using 10 μM proteins as described recently [35]. The results obtained from these measurements revealed no significant conformational changes (data not shown) between the monomeric and oligomeric protein species.

## Results

### PCS Study of AdR, Ad and CYP11A1

The aggregation states of AdR, Ad and CYP11A1 were tested by PCS. Data on photon correlation spectroscopy of AdR (5 μM) showed that the hydrodynamic diameter of AdR is *D* = (6 ± 2) nm, its content (β) constituting about 95 ± 5%. This value is similar to the appropriate value for AdR monomer from X-ray data (5.8 × 5.4 × 4.0 nm) [7].

According to X-ray data, the size of Ad (3.8 × 3.4 × 4.4 nm) is smaller than that of AdR. Since the intensity of relay scattering is proportional to D^6 ^[36], Ad concentration must be higher than the AdR one for obtaining the same PCS signal. Therefore, PCS procedure for Ad was carried out at a higher concentration (0.2 mM). The data obtained in the course of PCS studies show that the diameter of Ad particles is (5 ± 1) nm, their content (β) constituting about 100%. This value is similar to the one obtained for Ad monomer from X-ray data [37].

The PCS of cytochrome CYP11A1 (5 μM) was performed before and after the monomerization procedure. The particles with sizes (16 ± 2) nm were found in the absence of Emulgen 913 in the incubation mixture, their content (β) constituting 95 ± 5%. With addition of Emulgen 913 at the concentration 4–12%, the particle size decreases to (7 ± 2) nm. This was taken to mean that incubation in Emulgen solution leads to deaggregation of cytochrome CYP11A1. At the same time, the PCS analysis did not reveal the dependence of CYP11A1 deaggregation on Emulgen concentration in the 4–12% concentration range. Therefore, it is impossible to establish whether the CYP11A1 deaggregation is deep enough, i.e. whether it is able to produce monomers, dimers or trimers: apparently, the sensitivity of the device is not sufficient to ascertain that deaggregation did occur in the mixture of these species. In order to obtain more exact information about CYP11A1 aggregation, another, more sensitive technique should be used. As is known, the sensitivity of AFM molecular detector is very high—at a single molecular level. Earlier, we have shown that the AFM detector is able to distinguish binary complexes from monomers and ternary complexes from dimers and monomers [18,20,21]. In this study, the AFM detector was used to control the monomerization procedure of CYP11A1 as well as to visualize and measure the sizes of single protein molecules and their complexes within CYP11A1 system.

### AFM Visualization of the Individual Molecules of AdR, Ad and CYP11A1

By using the AFM method, one can obtain objective information about molecule height, while its lateral size may be broadened due to the limited size of the microscope's probe [20,38]. Therefore, in this study, the protein height was taken to be the only criterion for estimation of its size. As has been shown in [18,20,21], AFM allows distinguishing monomers from protein complexes based on the height of AFM-visualized objects. Therefore, in a series of AFM experiments, the heights of imaged proteins were measured, and the distribution of protein images with height was built.

#### AFM of Non-Monomerized and Monomerized CYP11A1

**Figure 1:**
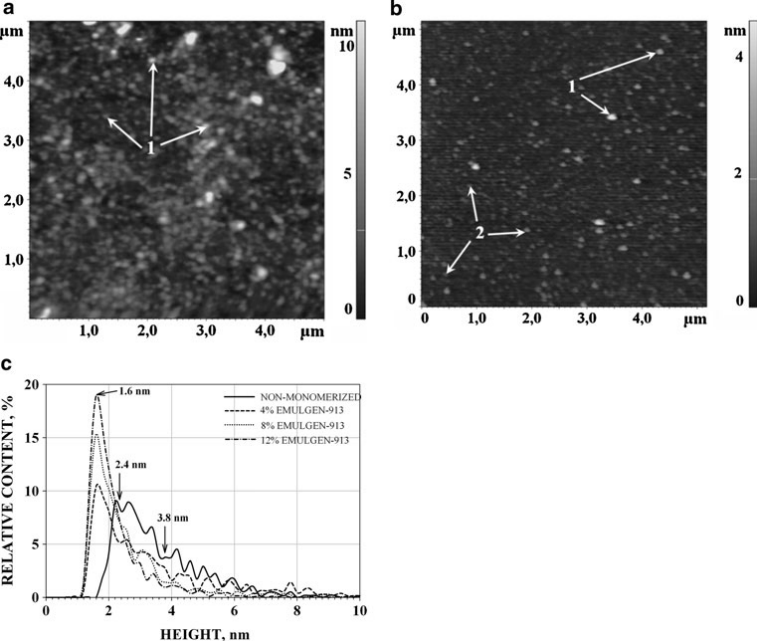
**AFM images of non-monomerized (a) and (12% Emulgen 913)-monomerized (b) CYP11A1 molecules and density of distribution (ρ(*h*)) with height of non-monomerized and monomerized CYP11A1 (c)**. Tapping mode. Experimental conditions were as follows: 100 μM CYP11A1 non-monomerized and 100 μM CYP11A1 monomerized in 50 mM KP, pH 7.4, containing Emulgen 913 (12%). For AFM visualization, the samples were diluted to obtain 1 μM CYP11A1 in 50 mM KP with 0.5% Emulgen 913, pH 7.4, and immediately placed onto the mica surface. *T* = 25°C. *Arrows (1)* indicate the images of CYP11A1 aggregates, *arrows (2)* indicate the images of CYP11A1 monomers.

AFM was used for visualization of non-monomerized and monomerized CYP11A1. The distribution densities ρ(*h*) of CYP11A1 images at 0, 4, 8 and 12% Emulgen 913 were obtained. The AFM images of oligomeric CYP11A1, which was not subjected to monomerization procedure (0% Emulgen 913), are presented in Figure 1a. Distribution of visualized species with height ρ(*h*) for each type of CYP11A1 was built (Figure 1c). This distribution is characterized by the position of the maximum near 2.4 nm and a broad width of the peak at the half-height (about 2 nm). The distribution was well approximated by the sum of two curves: ρ_1_(*h*) and ρ_2_(*h*) according to Eq. (2). Presented in Table 1 are the heights for which the maxima of appropriate distributions are observed at (*h*_max_)_1_ = 2.4 ± 0.3 nm and (*h*_max_)_2_ = 3.8 ± 0.4 nm.

**Table 1:** The AFM heights (*h*_max_) of distribution maximum of CYP11A1 images and the deaggregation degree (α) upon Emulgen 913 monomerization

Emulgen 913 concentration, %	*h*_max1_, nm	*h*_max2_, nm	% of monomers, α
CYP11A1			
0	2.4 ± 0.3	3.8 ± 0.4	0
4	1.6 ± 0.2	2.8 ± 0.2	45 ± 4
8	1.6 ± 0.2	2.8 ± 0.2	70 ± 4
12	1.6 ± 0.2	2.6 ± 0.2	82 ± 4

α = *N*/*N*_tot_ is the AFM-measured deaggregation degree, where *N* is the number of molecules with the 1.6 ± 0.2 nm diameter; *N*_tot_ is the total number of molecules

The AFM images of CYP11A1, subjected to monomerization procedure (12% Emulgen 913), are displayed in Figure 1b. Upon incubation of CYP11A1 in 4–12% Emulgen 913, the height maximum of AFM images was found to be decreased to *h*_max_ = 1.6 nm (Figure 1c). This was taken to mean that the incubation of CYP11A1 in 4–12% Emulgen 913 leads to deaggregation of this protein. Approximation of the distribution ρ(*h*) may be represented as the sum of two distributions: ρ_1_(*h*) with (*h*_max_)_1_ = 1.6 ± 0.2 nm and ρ_2_(*h*) with (*h*_max_)_2_ = 2.6–2.8 nm, calculated from Eq. (2) (Table 1). For each image of CYP11A1, incubated in 4, 8 and 12% Emulgen 913 solutions, ρ_1_(*h*) has the maximum (*h*_max_)_1_ = 1.6 ± 0.2 nm. Based on the fact that the monomers of P450 2B4 have the size 2.2 ± 0.2 nm [21] while Mr (P450 2B4) ≈ Mr (CYP11A1), it may be suggested that CYP11A1 images with the ρ_1_(*h*) maximum at *h*_max1_ = 1.6 ± 0.2 nm correspond to the monomers of CYP11A1. The AFM images of CYP11A1 monomers are represented in Figure 1b. The ρ_2_(*h*) curve with the (*h*_max_)_2_ ~ 2.6–2.8 nm corresponding to aggregates is consistent with the distribution of heights of oligomers with a varying degree of CYP11A1 aggregation. The deaggregation degree α = ρ_1_(*h*)/{(ρ_1_(*h*) + ρ_2_(*h*)} may be used for estimation of the share of deaggregated CYP11A1. With increasing Emulgen 913 concentration from 4 to 12%, the share of monomers was increased from 45 ± 4% to 82 ± 4% (Table 1).

Supersharp AFM analysis was used for additional confirmation of CYP11A1 monomerization by measuring of volumes of monomerized CYP11A1. The standard probe tip (*R* ~ 10–20 nm) broadening effect leads to substantial overestimation of measured protein's volume. At the same time, application of supersharp AFM probes allows to measure protein volume more correctly.

**Figure 2:**
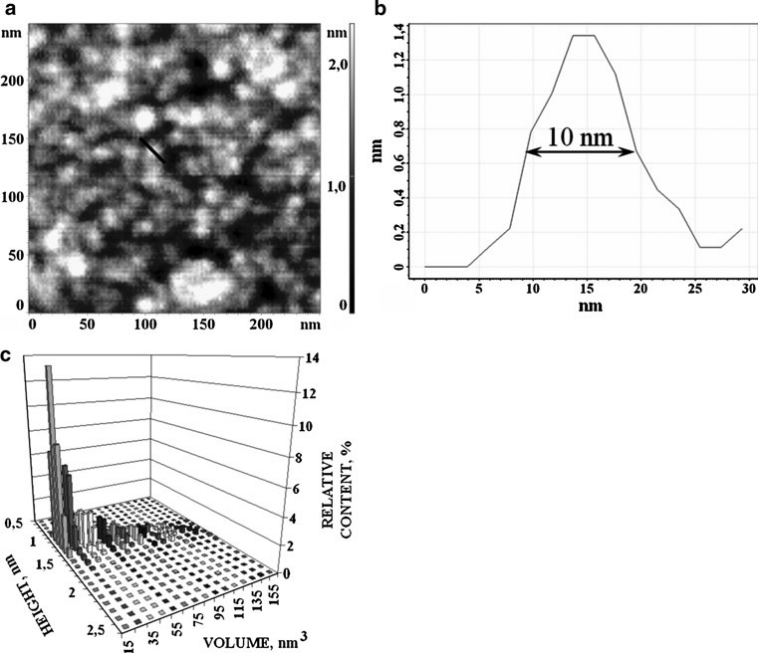
**a AFM image of monomerized CYP11A1 obtained using ultrasharp AFM probe; b cross-section, shown in (a); c density of distribution with height and volume of imaged CYP11A1**. Experimental conditions were as follows: 100 μM CYP11A1 monomerized in 50 mM KP, pH 7.4, containing Emulgen 913 (12%). For AFM visualization, the samples were diluted to obtain 1 μM CYP11A1 in 50 mM KP with 0.5% Emulgen 913, pH 7.4, and immediately placed onto the mica surface. *T* = 25°C. Tapping mode. AFM cantilevers were NSG01_DLC (NT-MDT, Russia).

Presented in Figure 2a are the images of adsorbed-on-mica monomerized CYP11A1 obtained by AFM with supersharp probes (*R* = 2 nm). Distribution of images with heights and volumes ρ(*h*, *V*) calculated from Eq. (3) is presented in Figure 2c. Objects, corresponding to this distribution, may be conventionally divided into 2 groups: (1) objects with volumes in the interval 15–45 nm^3^, with *V*_max_ = 15 ± 4 nm^3^, corresponding to *h*_max_ = 1.2 nm—distribution maximum of objects with heights in the interval *h* = 1.0–2.0 nm; (2) objects with volumes in the interval 55–155 nm^3^, with *V*_max_ = 55 ± 10 nm^3^, with heights in the interval *h* = 1.0–2.0 nm, *h*_max_ = 1.4 ± 0.1 nm.

Comparison of volumes *V*_max_ of AFM-imaged objects in group (1) with the volumes of truncated P4502B4 monomers (~30 nm^3^) from X-ray data [39] shows that objects with minimal sizes, i.e. those residing in group (1), correspond to CYP11A1 monomers accounting for 70% ± 10% of the total number of objects. Lateral sizes of imaged CYP11A1 monomers were in the order of 8–12 nm, with the most probable value ~10 nm.

Objects in group 2 with the volume *V*_max_ being twice larger and more than that of monomers apparently correspond to imaged dimers and oligomers of higher order accounting for 30 ± 10%.

The height of group (1)-imaged objects corresponding to monomers has the value of *h*_max_ = 1.2 ± 0.1 nm, which is essentially (twice) less than the height of P4502B4 from X-ray data (2.5 nm).

The lowered value of CYP11A1 height may be suggested to be due to the motility of the CYP11A1 molecule under the supersharp probe force or to the spreading of CYP11A1 molecules or else to their shrinkage by AFM probe or some other yet unknown causes.

Thus, AFM with supersharp probes also showed that CYP11A1 becomes monomeric upon monomerization procedure.

Naturally, the question arises as to whether the activity of CYP11A1 was retained after monomerization. In order to examine the functionality of CYP11A1 after monomerization with Emulgen 913, we performed in vitro CYP11A1 substrate conversion assays according to «Materials and Methods» (the "Control of functionality of monomeric CYP11A1" subheading). The results of these experiments have shown that the monomeric cytochrome is capable of converting 7-dehydrocholesterol to 7-dehydropregnenolone with *V*_max_ = 0.48 ± 0.02 nmol/min/nmol CYP11A1 and *K*_*M*_ = 0.32 ± 0.06 M. The *V*_max_ values and the Ad-dependent *K*_*M*_ values determined using monomeric CYP11A1 did not reveal any significant differences compared to the oligomeric enzyme for which these values were as follows: *V*_max_ = 0.51 ± 0.04 nmol/min/nmol CYP11A1 and *K*_*M*_ = 0.47 ± 0.15 M. Thus, the activity assays clearly demonstrate that the monomerization procedure does not significantly alter the functionality of CYP11A1.

#### AFM of AdR and Ad

**Figure 3:**
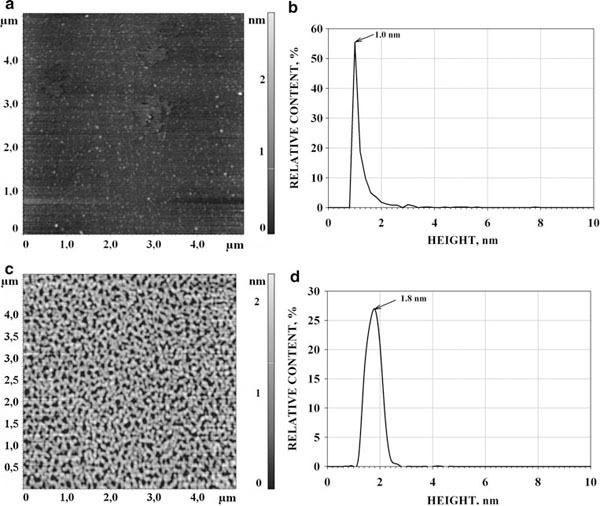
**AFM image (a) and density of distribution with height (b) of Ad; AFM image (c) and density of distribution with height (d) of AdR**. Tapping mode. Experimental conditions were as follows: 5 μl of 1 μM Ad and 1 μM AdR in 50 mM KP, pH 7.4 were deposited onto the freshly cleaved mica surface, *T* = 25°C.

Visualization of the AdR and Ad molecules was carried out as described in «Materials and Methods». AFM images of Ad and AdR on the mica surface were obtained (Figure 3a and 3c, respectively), and heights of the detected species were measured; also, the distribution of the number of visualized species with height ρ(*h*) for each type of measurements was built (Figure 3b, and 3d, respectively). The analysis of distributions for Ad (Mr = 13 kDa) shows that the majority of molecules (about 90%) have the height of about 0.8–1.8 nm (Figure 2b), with the ρ(*h*)_Ad_ maximum at *h*_max_ = 1.0 ± 0.2 nm < calculated from Eq. 2. Bearing in mind that the Mr_Ad_ (13 kDa) < Mr_AdR_ (50 kDa), it was inferred that the objects with the ρ(*h*) maximum at *h*_max_ = 1.0 ± 0.2 nm (Figure 3b) are Ad monomers. The analysis of distributions for AdR shows that the majority (about 90%) of molecules have the height of about 1.4–2.2 nm (Figure 3d), with the height maximum (*h*_max_) that corresponds to ρ(*h*)_AdR_ maximum at 1.8 ± 0.2 nm. Given that the AFM image of CYP11A1 monomer has the *h*_max_ = 1.6 ± 0.2 and the masses of AdR monomer (Mr = 50 kDa) and CYP11A1 monomer (Mr = 58 kDa) are similar, it may be suggested that the objects with the *h*_max_ = 1.8 ± 0.2 nm corresponds to AdR monomers. Thus, AdR species occurs predominantly in a monomeric form. The fact that the height of AdR is 2 times less than the one obtained from X-ray studies (4 nm) is probably explained by the molecule's distortion due to the probe force [18,40].

### AFM Investigation of Interactions Between Proteins Within CYP11A1 System

#### Ad/CYP11A1 Interaction

**Figure 4:**
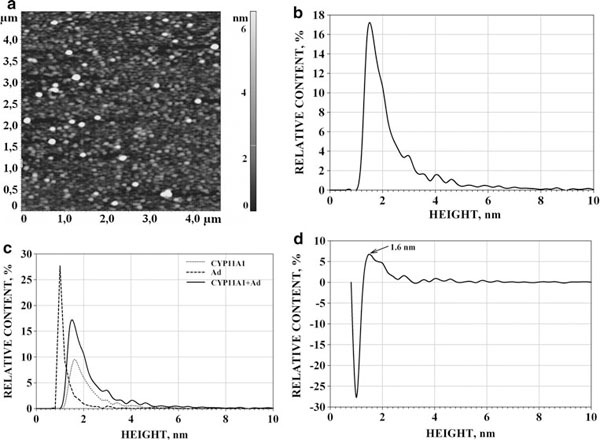
**AFM images of the objects (a) and the corresponding density of distribution with height (ρ(*h*)_Ad + CYP11A1_) for Ad + CYP11A1 mixture (b); comparison of ρ(*h*)Ad + CYP11A1 vs. normalized distribution densities of individual Ad and CYP11A1 monomers (summarized area under ρ(*h*)Ad and ρ(*h*)CYP11A1 curves is reduced to 100%) (c); differential curve (Δρ) between ρ(*h*)Ad + CYP11A1 and the sum of normalized distribution densities of individual Ad and CYP11A1 (d)**. Tapping mode. Experimental conditions were as follows: the mixture of 5 μM solutions (10 μl each) of appropriate individual proteins (monomeric CYP11A1, containing 1% Emulgen 913, and Ad) in 50 mM KP, pH 7.4, was incubated for 10 min, diluted 2.5 times with the same buffer, and a 5-μl portion of the mixture was immediately placed onto mica, *T* = 25°C.

The series of AFM experiments were carried out to investigate the interaction between Ad and CYP11A1. The images and the ρ(*h*) distribution for the imaged objects in the (CYP11A1_mon_ + Ad) mixture are presented in Figure 4a, b. Comparison of the (CYP11A1 + Ad) mixture distribution vs. Ad and CYP11A1 monomers' distributions (ρ(*h*)_Ad_ and ρ(*h*)_CYP11A1_) is presented in Figure 4c. The differential distribution (Δρ) between ρ(*H*)_CYP11A1mon+Ad_ distribution and ρ(*h*) distributions of individual CYP11A1_mon_ and Ad was calculated and represented in Figure 4d. One can see from Figure 4d that *h*_max_ for objects in the mixture is equal to that of CYP11A1 monomers. So this indicates the absence of other objects with different *h*_max_ in the mixture. Since the criterion chosen for distinguishing complex from monomer is based on comparison of distribution maximums, it may be concluded that in AFM experiments little or no Ad/CYP11A1_mon_ complex formation took place. Virtual lack of Ad/CYP11A1_mon_ complexation is possibly due to weak adhesion of Ad/CYP11A1_mon_ complexes to the AFM support—which in turn may be explained by blockage of adhesion sites of isolated Ad and CYP11A1_mon_ upon their complex formation.

At the same time, we have made an attempt to reveal the Ad/CYP11A1_mon_ complex formation by the plasmon resonance method. The BIAcore experiments enabled to register complex formation between CYP11A1 and Ad in the same conditions in which AFM experiments were conducted (see «Materials and methods» section). Based on the results of these experiments, the *k*_on_, *k*_off_ and *K*_*D*_ values for the Ad/CYP11A1_mon_ complex formation reaction were estimated as (290 ± 30) × 10^3^M^-1^ s^-1^, 0.05 ± 0.005 s^-1^ and 0.17 ± 0.015 μM, respectively (see Table 2). For the oligomeric enzyme, these values were as follows: *k*_on_ = (420 ± 40) × 10^3^ M^-1^ s^-1^, *k*_off_ = 0.09 ± 0.009 s^-1^ and *K*_*D*_ = 0.21 ± 0.02 μM (see Table 2). As seen from Table 2, there are no significant differences in the binding kinetics of the monomeric and oligomeric CYP11A1 with Ad: the *K*_*D*_ values varied by less than half.

**Table 2:** The values of *k*_on,_*k*_off_ and *K*_*D*_ for the Ad/CYP11A1 monomeric and the Ad/CYP11A1 oligomeric complex formation reaction

	*k*_on_ × 10^3^ [M^-1^ s^-1^]	*k*_off_ [s^-1^]	*K*_*D*_ [μM]
Monomeric CYP11A1	290 ± 30	0.05 ± 0.005	0.17 ± 0.015
Oligomeric CYP11A1	420 ± 40	0.09 ± 0.009	0.21 ± 0.02

Optico-biosensoric experiments were performed using a Biacore 3000 device. Approximately 400 RU of Ad were covalently immobilized on a carboxymethylated dextran matrix. Subsequently, different concentrations of CYP11A1 were passed over the flow cell. *K*_*D*_ values were determined using the 1:1 binding mechanism available in the Biacore evaluation software 4.1

Summarizing these results and the results on CYP11A1_mon_ activity determination (see part 2.1), it may be concluded that monomerized CYP11A1 can form complexes with Ad, at the same time CYP11A1 functionality was not affected by our monomerization procedure.

#### AdR/Ad Interaction

**Figure 5:**
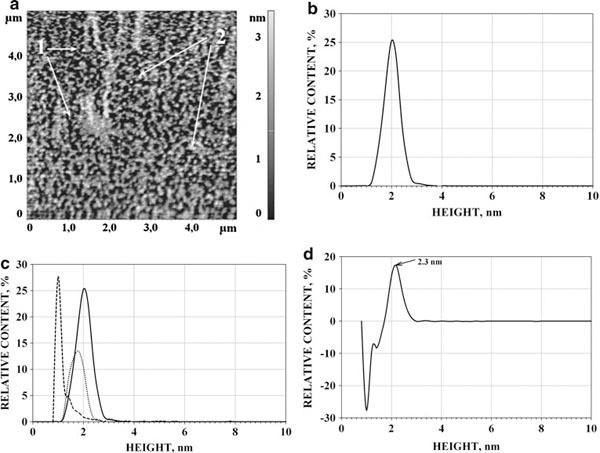
**AFM images (a) and the corresponding density of distribution with height (ρ(*h*)) for AdR/Ad complexes (b); comparison of ρ(*h*)_AdR/Ad_ vs. normalized distribution densities of individual AdR and Ad (the summarized area under ρ(*h*)_AdR_ and ρ(*h*)_Ad_ curves is reduced to 100%) (c); and the sum of normalized distribution densities of individual AdR and Ad (d)**. Tapping mode. Experimental conditions were as follows: the mixture of 5 μM solutions (10 μl each) of appropriate individual proteins (AdR and Ad) in the 50 mM KP, pH 7.4, was incubated for 10 min, diluted 2.5 times with the same buffer, and a 5-μl portion of the mixture was immediately placed onto mica, *T* = 25°C. *Arrows (1)* indicate the images of AdR and Ad monomers. *Arrows (2)* indicate the AdR/Ad images.

Binary AdR/Ad complexes were formed as described in «Materials and Methods». The images and the ρ(*h*) distribution for the imaged objects in the (AdR + Ad) mixture are presented in Figure 5a, b. Comparison of the (AdR + Ad) mixture distribution vs. the AdR and Ad monomers' distributions (ρ(*h*)_AdR_ and ρ(*h*)_Ad_) is presented in Figure 5c. The differential distribution (Δρ) between ρ(*h*)_AdR+Ad_ distribution and distributions of individual AdR and Ad was calculated and represented in Figure 5d.

This Δρ = (ρ(*h*)_AdR+Ad_ - [ρ(*h*)_AdR_ + ρ(*h*)_Ad_]) is characterized with the new height maximum at *h*_max_ = 2.3 ± 0.2 nm in its positive wing (see Figure 5d). This *h*_max_ is higher than the *h*_max_ = 1.8 nm (AdR) or the *h*_max_ = 1.0 nm (Ad). Therefore, in contrast to the case with the (CYP11A1mon + Ad) mixture, the AFM height distribution for the (AdR + Ad) mixture is characterized by the appearance of some objects with heights in a range 1.8–2.6 nm and with higher *h*_max_ than the ones of individual AdR and Ad. In the binary mixture, the share of these objects in the positive wing of the Δρ = (ρ(*h*)_AdR+Ad_ - [ρ(*h*)_AdR_ + ρ(*h*)_Ad_]) distribution with the height 1.8–2.6 nm reached (51 ± 8)%. Appearance of the positive wing allows us to conclude that the increase in the number of objects with the height 1.8–2.6 nm and the hmax at 2.3 ± 0.2 nm up has been due to formation in the (AdR + Ad) mixture of binary AdR/Ad complexes (Table 3).

**Table 3:** AFM-measured heights of protein and protein complexes heights in CYP11A1 system

Name of protein or complex	AFM-measured object heights, nm
CYP11A1 monomer (*M*_*r*_ = 56 kDa)	1.4–2.8 with *h*_max_ = 1.6 ± 0.2
AdR monomer (*M*_*r*_ = 60 kDa)	1.4–2.2 with *h*_max_ = 1.8 ± 0.2
Ad monomer (*M*_*r*_ = 16 kDa)	0.8–1.8 with *h*_max_ = 1.0 ± 0.2
Ad + CYP11A1	1.3–2.6 with *h*_max_ = 1.6 ± 0.2
AdR/Ad	1.8–2.6 with *h*_max_ = 2.3 ± 0.2
AdR/CYP11A1	2.2–5.0 with *h*_max_ = 2.8 ± 0.2
AdR/Ad/CYP11A1	2.8–5.5 with *h*_max_ = 4.0 ± 1.0

#### AdR/CYP11A1 Interaction

**Figure 6:**
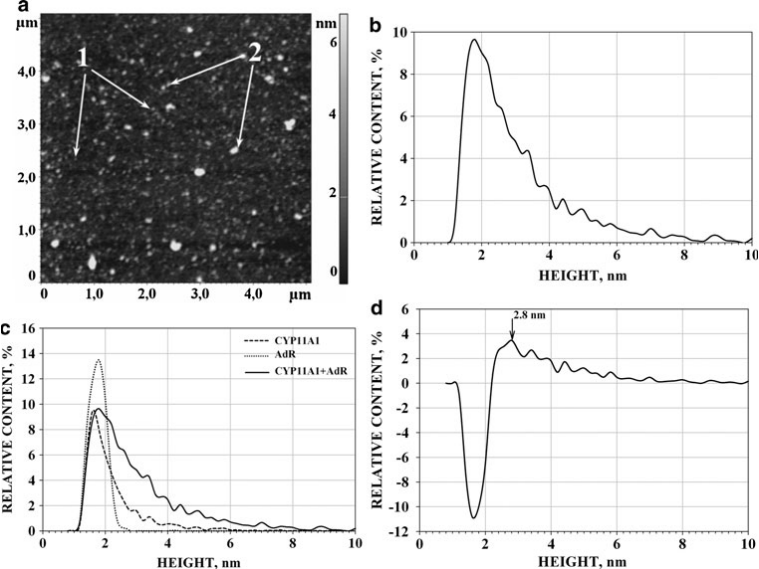
**AFM images (a) and the corresponding density of distribution with height (ρ(*h*)) for AdR/CYP11A1 complexes (b); comparison of ρ(*h*)_AdR/CYP11A1_ distribution vs. normalized distribution densities of individual AdR and CYP11A1_mon_ (the summarized area under ρ(*h*)_CYP11A1_ and ρ(*h*)_AdR_ curves is reduced to 100%) (c); differential curve (Δρ) between ρ(*h*)_CYP11A1/AdR_ for mixture and the sum of normalized distribution densities of individual CYP11A1_mon_ and AdR (d)**. Tapping mode. Experimental conditions were as follows: the mixture of 5 μM solutions (10 μl each) of appropriate individual proteins (monomeric CYP11A1, containing 1% Emulgen 913, and AdR) in the 50 mM KP, pH 7.4, was incubated for 10 min, diluted 2.5 times in the same buffer, and a 5-μl portion of the mixture was immediately placed onto mica, *T* = 25°C. *Arrows (1)* indicate the images of AdR and CYP11A1 monomers. *Arrows (2)* indicate the AdR/CYP11A1 images.

The similar situation to the above-described one was met for imaged objects in the (AdR + CYP11A1_mon_) mixture (Figure 6). The ρ(*h*) of imaged objects is represented in Figure 6b. Comparison of distribution for the (AdR + CYP11A1_mon_) mixture vs. the distributions of individual AdR and CYP11A1_mon_ is shown in Figure 6c. As in the case with the (AdR +Ad) mixture, the differential curve of distributions Δρ = (ρ(*h*)_AdR+CYP11A1_ - [ρ(*h*)_AdR_ + ρ(*h*)_CYP11A1_]) presented in this study is characterized by the appearance of the positive wing of distribution of objects with heights 2.2–5.0 nm and *h*_max_ = 2.8 ± 0.2 nm (see Figure 6d). This *h*_max_ is higher than the *h*_max_ = 1.6 nm (CYP 11A1^mon^) or the *h*_max_ = 1.8 nm (AdR). In the binary mixture, the share of these new objects in the positive wing of the differential spectrum Δρ = (ρ(*h*)_AdR+CYP11A1_ - [ρ(*h*)_AdR_ + ρ(*h*)_CYP11A1_]) with heights 2.2–5.0 nm reached (35 ± 7)%. Based on these data, it was concluded that the increase in the number of objects with heights 2.2–5.0 nm and the *h*_max_ = 2.8. ± 0.2 nm up has been due to the formation in the (AdR + CYP11A1_mon_) mixture of binary AdR/CYP11A1 complexes (Table 3).

#### AdR/Ad/CYP11A1 Interaction

**Figure 7:**
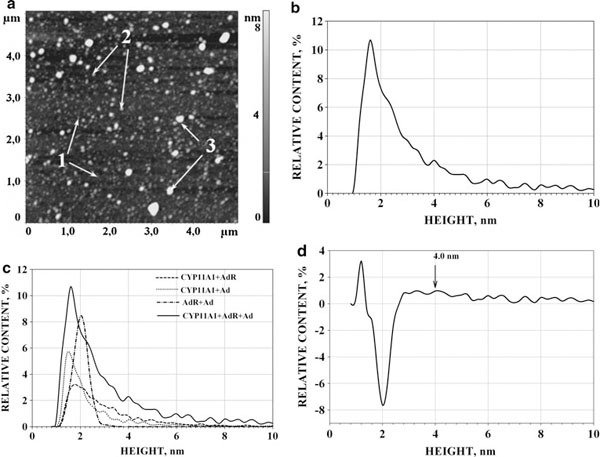
**AFM image (a) and the corresponding density of distribution with height ρ(*h*)) for CYP11A1/AdR/Ad (b); comparison of ρ(*h*)_AdR/Ad/CYP11A1_ distribution vs. normalized ρ(*h*)_CYP11A1/Ad_, ρ(*h*)_CYP11A1/AdR_ and ρ(*h*)_AdR/Ad_ distributions (the summarized area under curves ρ(*h*) for binary mixtures is reduced to 100%) (c); differential curve (Δρ) between ρ(*h*)_CYP11A1/AdR/Ad_ for ternary mixture and the sum of ρ(*h*)_CYP11A1/Ad_, ρ(*h*)_CYP11A1/AdR_ and ρ(*h*)_AdR/Ad_ for binary mixtures (d)**. *Arrows (1)* indicate the images of protein monomers. *Arrows (2)* indicate the images of the binary protein complexes. *Arrows (3)* indicate the images of the ternary CYP11A1/AdR/Ad complexes. Tapping mode. Experimental conditions were as follows: mixture of 7.5 μM solutions (10 μl each) of appropriate individual proteins (monomeric CYP11A1, containing 1.5% Emulgen 913, Ad and AdR) in 50 mM KP, pH 7.4, was incubated for 10 min, diluted 2.5 times in the same buffer, and a 5-μl portion of the mixture was immediately placed onto mica.

While in our earlier optico-biosensoric studies the formation of ternary CYP11A1_nonmonomerized_/Ad/AdR complexes was merely registered [10], in the present research the AFM visualization of the (CYP11A1_mon_ + Ad + AdR) mixture was accomplished (see Figure 7); the ρ(*h*) distribution obtained upon analysis of imaged objects (Figure 7b) was compared with the three distributions for the binary mixtures: ρ(*h*)_CYP11A1+Ad_, ρ(*h*)_Ad+AdR_ and ρ(*h*)_AdR+CYP11A1_. This comparison of the distribution of the ρ(*h*)_CYP11A1+Ad+AdR_ for the (AdR + Ad + CYP11A1_mon_) mixture vs. ρ(*h*)_CYP11A1+Ad_, ρ(*h*)_Ad+AdR_ and ρ(*h*)_AdR+CYP11A1_ is shown in Figure 7c and the differential curve Δρ = [ρ(*h*)_CYP11A1+Ad+AdR_ - $\sum_{i=1}^{3}$ ρ_*i*_(*h*)_BINARY MIXTURE_] is represented in Figure 7d. This distribution is characterized by the appearance of the positive wing of differential distribution Δρ of objects with heights 2.8–5.5 nm and a broad maximum at the *h*_max_ = 4.0 ± 1.0 nm in the differential curve Δρ = [ρ(*h*)_CYP11A1+Ad+AdR_ - $\sum_{i=1}^{3}$ ρ_*i*_(*h*)_BINARY MIXTURE_]. The share of these objects in the 3-component mixture was (12 ± 4) %. Based on these data, it was inferred that the majority of objects with maximum at *h*_max_ = 4.0 ± 1.0 nm are, in fact, the ternary d-CYP11A1/Ad/AdR complexes (Table 3). It is to be noted that formation of ternary d-CYP11A1/Ad/AdR complexes (as well as formation of binary AdR/Ad and AdR/CYP11A1 complexes) was demonstrated in our earlier optico-biosensoric studies [10,11].

## Conclusion

Thus, atomic force microscopy (AFM) was used in this work to detect and visualize the isolated proteins and protein complexes between the components of the monomeric CYP11A1-dependent steroid hydroxylase system. For this purpose, at the first step the procedure for cytochrome CYP11A1 monomerization in solution was developed, and the control of the monomerization degree based on the AFM and PCS methods was established. It was shown that the incubation of CYP11A1 with 12% Emulgen leads to the dissociation of aggregates to monomers with the monomerization degree of (82 ± 4) %. The Ad, AdR and CYP11A1 images were obtained, and their heights were measured. It was found that the AFM is able to identify and visualize not only the individual membrane-bound proteins but also the binary Ad/AdR, AdR/CYP11A1 and the ternary Ad/AdR/CYP11A1 complexes within the CYP11A1-containing hydroxylase system. In addition, it was shown that the CYP11A1 monomerization procedure developed in this study did not influence the functionality of the cytochrome. In conclusion, the AFM technique provides a valuable tool for the complex formation studies particularly for the analysis of complexes that involve membrane-bound proteins such as CYP11A1. Moreover, application of AFM technology opens up possibilities for the revelation and investigation of other, yet unknown, protein complexes.
